# Subdiaphragmatic vagus nerve activity and hepatic venous glucose are differentially regulated by the central actions of insulin in Wistar and SHR

**DOI:** 10.14814/phy2.12381

**Published:** 2015-05-06

**Authors:** Izabela Martina R Ribeiro, Hildebrando C Ferreira-Neto, Vagner R Antunes

**Affiliations:** Department of Physiology and Biophysics, Institute of Biomedical Sciences, University of Sao Paulo (USP)Sao Paulo, Brazil

**Keywords:** Autonomic nervous system, hepatic venous glucose, insulin, lumbar splanchnic nerve, subdiaphragmatic vagus nerve

## Abstract

Glucose is the most important energy substrate for the maintenance of tissues function. The liver plays an essential role in the control of glucose production, since it is able to synthesize, store, and release glucose into the circulation under different situations. Hormones like insulin and catecholamines influence hepatic glucose production (HGP), but little is known about the role of the central actions of physiological doses of insulin in modulating HGP via the autonomic nervous system in nonanesthetized rats especially in SHR where we see a high degree of insulin resistance and metabolic dysfunction. Wistar and SHR received ICV injection of insulin (100 nU/*μ*L) and hepatic venous glucose concentration (HVGC) was monitored for 30 min, as an indirect measure of HGP. At 10 min after insulin injection, HVGC decreased by 27% in Wistar rats, with a negligible change (3%) in SHR. Pretreatment with atropine totally blocked the reduction in HVGC, while pretreatment with propranolol and phentolamine induced a decrease of 8% in HVGC after ICV insulin injection in Wistar. Intracarotid infusion of insulin caused a significant increase in subdiaphragmatic vagus nerve (SVN) activity in Wistar (12 ± 2%), with negligible effects on the lumbar splanchnic sympathetic nerve (LSSN) activity (−6 ± 3%). No change was observed in SVN (−2 ± 2%) and LSSN activities (2 ± 3%) in SHR after ICA insulin infusion. Taken together, these results show, in nonanesthetized animals, the importance of the parasympathetic nervous system in controlling HVGC, and subdiaphragmatic nerve activity following central administration of insulin; a mechanism that is impaired in the SHR.

## Introduction

Glucose is the most important energy substrate for the maintenance of tissues function. Variability in glucose disposal, either indiscriminate increase or reduction, causes cell dysfunction (Pilkis and Granner 1992). Given the importance of glucose to normal organ function, it is essential that plasma levels of glucose to be controlled in order to prevent large fluctuations (Nordlie et al. 1999).

The liver is an important organ for the homeostatic maintenance of blood glucose due to its ability to uptake and store glucose as glycogen when circulating levels are high, and to evoke the breakdown of glycogen and release glucose when blood glucose concentration is very low (Perseghin et al. 1997; Nordlie et al. 1999). Due to its pivotal role on physiological functions, glucose has to be precisely controlled by different mechanisms, which involve the actions of hormones, such as insulin, and liver function (Perseghin et al. 1997). Insulin is a potent anabolic hormone capable of controlling, among other functions, the uptake and maintenance of glucose availability (Aronoff et al. 2004). In this sense, it is important to understand the link between the action of insulin and hepatic production of glucose (HGP), and here we have measured the glucose level directly into the hepatic vein, named as hepatic venous glucose concentration (HVGC), as a predictor of availability of glucose to the circulation provided by the liver.

Besides it's peripheral action, there are evidences showing that the brain (more specifically, hypothalamus) is also sensitive to insulin (Baskin et al. 1983; Szabo et al. 1983; Obici et al. 2002; Gerozissis 2003; Pocai et al. 2005). It has been reported that intracarotid (ICA) injection of insulin may enter privileged sites within the CNS (such as the hypothalamus), and evoke a rapid decrease in HGP, resulting in a fall of the blood glucose concentration (Szabo and Szabo 1975). Moreover, it is known that a pretreatment with cholinergic receptors antagonist as well as bilateral vagotomy in anesthetized animals is able to block the hypoglycemia induced by insulin infusion within the CNS (Szabo and Szabo 1972, 1975). What still remains to be determined is how physiological doses of insulin acting on the CNS modulate the HVGC via autonomic nervous system in nonanesthetized animals, since anesthetic exert a great influence on sympathetic and parasympathetic balance (Göther 1982; Pearse 2002; Holscher et al. 2008). Thus, here we are proposing that central action of insulin at low doses would be more appropriate to control the hepatic glucose homeostasis, particularly in conscious animals, which is closer to the physiological condition.

Previous studies have demonstrated a nonuniform activation of sympathetic nerve activity in spontaneously hypertensive rats (SHR) and Wistar as a response to chronic peripheral infusion of high doses of insulin for long periods (Morgan et al. 1993). In addition, SHR features characteristics as insulin resistance (Hulman et al. 1993), hyperinsulinemia and changes in lipid profile, well characterized as a model of metabolic syndrome (Potenza et al. 2005). Here, we hypothesize that this metabolic dysfunction and autonomic imbalance observed in SHR, may compromise the central actions of insulin on HGVC, when compared to Wistar rats.

Therefore, the present investigation was undertaken to elucidate the direct central actions of insulin signaling on the rapid regulation of HGVC in Wistar and SHR. We used an integrated physiological approach in combination with pharmacological manipulation and electrophysiological recordings of the parasympathetic and sympathetic efferent nerves to the liver to determine the putative neural pathways that control HVGC in nonanesthetized rats.

## Methods

### Ethical approval

All experimental procedures were performed in accordance with the Ethical Principles in Animal Research of the Brazilian College of Animal Experimentation and were approved by the Ethical Committee for Animal Research of ICB/USP (Protocol #38/2010).

### Animals

Male Wistar rats and SHR (8–9 weeks old; ~250 g) were used for the in vivo studies, and 3–4 week old rats (50–80 g) for in situ studies (*n* = 44 in total). All animals were obtained from the colony bred at the Institute of Biomedical Sciences, University of Sao Paulo (ICB/USP), and housed in rooms with constant temperature of 22–24°C and a relative humidity of 50–60% under a controlled light/dark cycle (12/12 h) with normal rat chow (Nuvilab^®^) and drinking water ad libitum.

### Intracerebroventricular (ICV) microinjections

Rats were anesthetized with a mixture of ketamine (100 mg/kg, Syntecvet- Syntec) and xylazine (20 mg/kg, Syntecvet- Syntec) via intraperitoneal injection. After checking the depth of anesthesia by assessing limb withdrawal reflexes to noxious pinching and the absence of the blink reflex to corneal stimulation, the animals were placed in a stereotaxic head frame (Model 900; David Kopf, Tujunga, CA) with the incisor bar set at –3.3 mm below the interaural line. The skin overlying the skull was reflected back, bregma and lambda were positioned at the same horizontal level, and a 1-mm burr hole was drilled in the parietal bones according to coordinates derived from the atlas of Paxinos and Watson (2007). Unilateral stainless steel 23-gauge guide cannulas (13 mm of length) were implanted immediately above the lateral ventricle (LV) following the coordinates: −1 mm caudal to bregma, −1.6 mm lateral to midline, and −3.5 mm below the surface of the skull. The guide cannulae were fixed to the skull with dental acrylic resin and watch screws, and closed with a metallic occluder until the day of the experiments. At the end of the surgery, animals received an intramuscular injection of antibiotic (veterinary antibiotic 1%, 0.03 mL/rat, Fort Dodge Saúde Animal Ltda., Campinas, SP, Brazil) and a subcutaneous injection of analgesic/anti-inflammatory (ketoprofen 1%, 0.03 mL/rat, Ketoflex, Mundo Animal, SP, Brazil). Rats were allowed to recover for 4 days after the surgery before hepatic vein catheterization. All microinjections into LV were conducted in conscious freely moving rats. The needle (33 gauge, Small Parts, Miami Lakes, FL) used for microinjection into the LV was 1.0 mm longer than the guide cannula and was connected by PE-10 tubing to a 10-*μ*L syringe (Hamilton, Reno, NV). The needle was carefully inserted into the guide cannula and slow manual injections were performed 30 sec later. The volume injected was 1 *μ*L.

### Catheterization of the hepatic vein and femoral artery and cardiovascular recordings

One day before the experiments, under ketamine and xylazine anesthesia, a polyethylene catheter (PE-50, Clay Adams, Parsippany, NJ) of approximately 8 cm of length (length varied according to the animal size) was inserted into right jugular vein in direction to the hepatic vein for repeated blood samples collection and systemic administration of drugs. The catheter was then blocked at the free end with a metal pin and the confirmation of the catheter tip placement into the hepatic vein was verified visually and manually at the end of the experiments. Only animals with the catheter placed exactly into the hepatic vein were considered in the analysis. For cardiovascular recordings, a catheter (PE-10 connected to PE-50, Clay Adams, Parsippany, NJ) was inserted into the abdominal aorta through the femoral artery. All catheters were tunneled subcutaneously and exteriorized through the back of the neck and experiments were performed in conscious freely moving rats. Monitoring of pulsatile arterial pressure (PAP, mmHg), mean arterial pressure (MAP, mmHg), and heart rate (HR, bpm) were performed by connecting the arterial catheter to a pressure transducer (MLT844, ADInstruments, Sydney, NSW, Australia) coupled to a preamplifier (ML224 Quad Bridge, ADInstruments, New South Wales, Australia) connected to a data acquisition system-Powerlab running a LabChart software that provides data display, recording and analysis (ADInstruments, New South Wales, Australia). The data sampling and acquisition rate was 1000 Hz and changes on PAP (systolic and diastolic), MAP and HR were analyzed off-line.

### Monitoring of hepatic venous blood glucose in conscious rats

Blood samples were collected from the hepatic vein catheter in conscious freely moving rats after the animal had acclimatized to the experimental room. Samples of hepatic venous blood (one drop) were taken immediately before and 2, 5, 10, 20, and 30 min after the microinjection of insulin or denatured insulin into the LV. In another group, rats were pretreated with intravenous injection of atropine methyl-bromide and/or phentolamine + propranolol, depending on protocol) and hepatic blood samples were taken before and at 2, 5, 10, 20, 30, 40, 50, and 60 min after insulin ICV microinjection. HVGC was measured with a glucometer (Accu Check Active; Roche Diagnostics GmbH, Mannheim, Germany).

### Nerve recordings and intracarotid (ICA) injection of insulin

Nerve recordings were performed in a decorticate, nonanesthetized, arterially perfused in situ preparation of rat (DAPR) as previously described (Antunes et al. 2006). Briefly, rats were deeply anesthetized with halothane (5%) until loss of paw withdrawal reflex. The stomach, intestines, and spleen were ligated and removed via midline laparotomy and the whole liver was kept intact. The sternum was split and the ribcage retracted to allow access to the mediastinum. The pericardium was removed and the left phrenic nerve was isolated. The animal was submerged in cooled artificial cerebrospinal fluid (aCSF, see below) and the cerebral hemispheres exposed by removal of the parietal bones. The cerebral cortices, hippocampus and thalamic area were removed by gentle aspiration. The removal of these structures abrogates the need for further anesthetic use in this preparation. Care was taken to ensure the preoptic area and its adjacent septal nuclei and hypothalamic areas remained fully intact. The preparation was skinned and transferred to the recording chamber. A double-lumen perfusion cannula was inserted into the ascending aorta via the left ventricle. The preparation was perfused at flow rates of 28 ± 2 mL/min using a roller pump (Watson Marlow 505S, UK) with aCSF containing an oncotic agent (PEG 20,000, 1.5%; Sigma, St Louis, MO), gassed with carbogen (95% O_2_ and 5% CO_2_), warmed to 32°C and filtered using a nylon screen (pore size: 25 *μ*m). After respiratory-related movements commenced, a neuromuscular blocker (vecuronium bromide, 4 mg/mL, Vecuron, Cristália, SP, Brazil) was added to the perfusate to mechanically stabilize the preparation. The second lumen of the cannula was used to monitor aortic perfusion pressure.

The phrenic nerve (PN), lumbar sympathetic chain (L2–L3), and subdiaphragmatic vagus efferent nerves (SVN) were visualized through a binocular microscope and nerve activity recorded from their distal end using a glass suction bipolar electrode held in a 3-D micromanipulator. Rhythmic, ramping phrenic nerve activity (PNA) gave a continuous physiological index of preparation stability and viability. Preparations that did not show ramping PNA were deemed unviable and not included in the study. Signals were AC-amplified (NL104, Neurolog, UK) and band-pass filtered (100 Hz–3 kHz) and displayed on a computer using the Spike 2 software (Cambridge Electronic Design, Cambridge, UK). The lumbar splanchnic sympathetic nerve (LSSN) activity exhibited marked respiratory modulation, and was attenuated by an increase in perfusion pressure (arterial baroreceptor stimulation). The SVN was identified and dissected following displacement of the liver to the right of the animal. The ventral trunk of the SVN was cut distally close to origin of the common hepatic branch and multifiber nerve recordings were performed using a glass suction electrode. For evaluating the central effects of insulin, a single bolus (100 nU; injection volume of 200 mL) was injected into the double-lumen catheter in direction of the carotid arteries (200 mL of total circulating volume), simultaneously to LSSN and SVN recordings. At the end of the in situ studies, animals were terminally anesthetized with pentobarbital sodium (>60 mg/kg of body weight) added in the perfusion solution, and after nerves activities' cessation, the perfusion pump was turned off.

### Chemicals and solutions

We used a CSF containing (in mmol/L): NaCl 120, NaHCO_3_ 24, KCl 5, CaCl_2_ 2.5, MgSO_4_ 1.25, KH_2_PO_4_ 1.25, dextrose 10; Human Insulin, a polypeptide hormone produced by the *β*-cells of pancreatic islets (Lilly, Indianapolis, IN, EUA) for LV (100 nU/*μ*L) and intracarotid injection (100 nU); Atropine methyl-bromide (ATROP, 2 mg/kg), a competitive muscarinic receptor antagonist that does not cross the blood–brain barrier; Phentolamine (PHENT, 3 mg/kg), an *α*-adrenoceptor antagonist; Propranolol hydrochloride (PROP, 0.5 mg/kg), a *β*-adrenoceptor antagonist. The drug solutions were freshly dissolved in sterile saline (NaCl 154 mmol/L) and sodium bicarbonate was added to adjust the pH to 7.4 when necessary. All salts and drugs were purchased from Sigma-Aldrich (St Louis, MO) unless otherwise stated.

### Histological analysis

At the end of in vivo studies Evan's Blue dye (2% w/v) was microinjected into the LV to confirm proper cannula placement. Immediately after dye injection, the animals were deeply anesthetized with pentobarbital sodium (>60 mg/kg of body weight, ip) and transcardially perfused with sodium phosphate buffer (0.01 mol/L) followed by 4% paraformaldehyde. The brains were removed and kept in a sucrose solution (0.2 g/mL) in 4% paraformaldehyde for 48 h, then rapidly frozen with nitrogen liquid and cut in coronal sections (40 *μ*m of thickness) with a cryostat (CM 1950 Nussloch, Leica Microsystems, Wetzlar, Germany) and thaw-mounted on gelatin-subbed glass slides. Brain sections were visualized under light microscopy (darkfield) to identify the site of microinjection. Only animals that showed the spread of dye throughout the lateral ventricle and the track of the guide cannula into the LV were analyzed.

### Experimental protocol

Prior to the experiments animals were food deprived for a period of 12–14 h (overnight) but given free access to water. All experiments were performed in the morning (9–11 am) and commenced after complete acclimatization of animals to the experimental environment. HVGC was deemed as the glucose level measured from the venous blood collected directly from the hepatic vein of conscious rats, prior (basal levels) and immediate after insulin administration into the brain.

#### Effects of insulin injected into the LV on hepatic venous glucose concentration in conscious Wistar and SHR

Insulin (100 nU/*μ*L) was injected into the LV and the blood glucose from the hepatic venous catheter was measured in duplicate at time 0 (basal), 2, 5, 10, 20, and 30 min in conscious freely moving rats. In control experiments, insulin was denatured by immersing an aliquot in a sealed flask of stock solution into boiling water for 15 min. The denatured insulin was injected into the LV and hepatic glucose monitored over the same time course as described above.

#### Effects of autonomic neurotransmitter inhibitor agents on HVGC elicited by central actions of insulin in conscious Wistar and SHR

The action of autonomic outflow on glucoregulatory mechanisms elicited by microinjection of insulin into the LV was investigated by the prior administration of *α*-(phentolamine, 3 mg/kg) and *β*-(propranolol 0.5 mg/kg) adrenoceptor antagonists, and muscarinic receptors antagonist (atropine methyl-bromide, 2 mg/kg). These were injected into the hepatic vein of conscious freely moving rats while blood pressure and heart rate was simultaneously recorded. After administration of the autonomic antagonists, and their efficacy verified by changes in the basal levels of arterial blood pressure and heart rate (data not shown), animals received a LV injection of insulin (100 nU/*μ*L) and blood glucose from the hepatic venous catheter measured at 2, 5, 10, 20, and 30 min.

#### Effects of intracarotid injection of insulin on subdiaphragmatic vagus nerve (SVN) and lumbar splanchnic sympathetic nerve (LSSN) activities of Wistar and SHR

The SVN and LSSN have known innervation patterns to the gastrointestinal tract and liver (Berthoud et al. 1992; Phillips et al. 1997). For this reason, we investigated the effects of intracarotid insulin injection on the activities of both the SVN and LSSN, which is considered a good index of the sympatho-vagal balance related to putative hepatic venous glucose concentration elicited by the central actions of insulin. The effects of insulin on SVN and LSSN activities were monitored for 30 min after a single intracarotid injection. At the end of each experiment the remaining LSSN and SVN activity was assessed by application of hexamethonium into the perfusate (5 mmol/L) and turning the perfusion pump off, respectively. The background noise was then subtracted from nerves signals during data analysis.

### Statistical analysis

Statistical analyses were performed by using commercial software (GraphPad Prism 4.02, GraphPad Software Inc., CA). Hepatic venous glucose concentration and changes in cardiovascular parameters were expressed as percentage change over baseline. All data are expressed as mean ± standard error mean (SEM) and were statistically analyzed by a one-way ANOVA followed by Bonferroni's post hoc test, and the baseline data by an unpaired *t*-test. Nerve recording data were acquired using biopotential AC amplifiers and filters (Neurolog, Digitimer Ltd, UK) and collected using a CED 1401 A–D interface (CED, Cambridge Electronic Design, Cambridge, UK) and a computer running Spike 2 software (CED) with custom-written scripts for data acquisition and on- and off-line analyses. LSSN activity was displayed as a moving average (100 msec or 2 sec time constant, depending on the protocol used). To standardize the data across preparations, changes in LSSN and SVN activity were expressed as a percentage of the basal values. To analyze the time course changes in the SVN and LSSN activities evoked by intracarotid injection of insulin a cursor was positioned 2 min before insulin infusion and a second cursor was positioned at the exact moment of insulin application. This period was designated the basal nerve activity. All subsequent nerve activity were measured every 2 min for 30 min. Statistical analysis was also performed on values obtained from measuring area under the curve (AUC) from the HVGC data obtained in in vivo studies and in the SVNA and LSSNA data from the in situ studies. One-way ANOVA for repeated measures followed by Bonferroni's post hoc test was used. The results are expressed as mean ± standard error mean (SEM) and “*n*” represents the number of animals per group. Statistical significance levels were set at *P *≤* *0.05.

## Results

### Intracerebroventricular insulin administration decreases hepatic venous glucose concentration in Wistar but not in SHR

**Figure 1:**
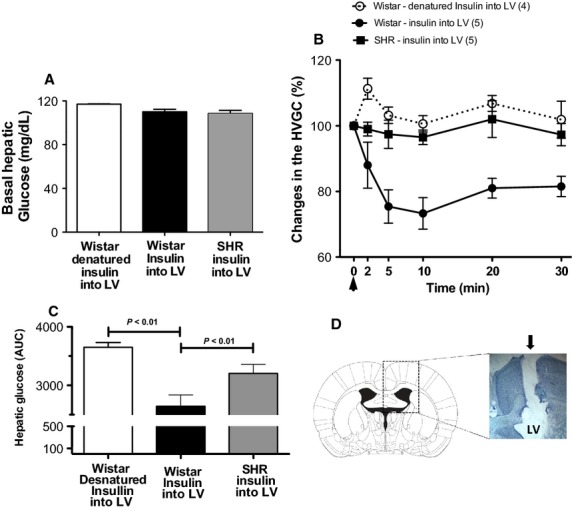
Effects of central insulin action on hepatic venous glucose concentration (HVGC) in nonanesthetized Wistar and SHR. (A) Basal levels of hepatic glucose (mg/dL) prior to injection of insulin and/or denatured insulin into the lateral ventricle (LV) of Wistar (*n* = 5) and SHR (*n* = 5). (B) Percentage (%) change in HVGC and area under the curve (C) after administration of insulin and/or denatured insulin (vehicle control) into the LV (arrow) of Wistar and SHR. (D) Schematic diagram of coronal section of the rat brain modified from the Atlas of Paxinos and Watson (2007) and a representative photomicrograph of a sagittal brain section in a higher magnification showing the injection track (arrow) of insulin and/or denatured insulin into the lateral ventricle (LV) of one representative animal. (*n*) Numbers of animals of each group; One-Way ANOVA;* P* < 0.01.

To determine whether centrally administered insulin can elicit changes in the HVGC, we performed microinjections of insulin or denatured insulin (vehicle control) into the LV (Fig.1D) and simultaneously monitored the HVGC in conscious freely moving Wistar and SHR. Before the onset of experiments the basal HVGC level was measured and did not differ between Wistar (110 ± 5 mg/dL, *n* = 5) and SHR (108 ± 7 mg/dL, *n* = 5, Fig.1A). Figure1B illustrates the changes in HVGC after microinjection of insulin or denatured insulin into the LV of conscious Wistar and SHR. In Wistar rats, the hepatic venous glucose level decreases immediately at 5 (84 ± 8 mg/dL) and 10 min (81 ± 8 mg/dL) post-ICV insulin administration, and started to return to baseline levels at 20 (89 ± 6 mg/dL) and 30 min (90 ± 7 mg/dL). In contrast, in SHR group microinjection of insulin into the LV failed to elicit any significant change in the hepatic glucose levels over the same time course: 5: (106 ± 9 mg/dL), 10: (105 ± 7 mg/dL), 20: (109 ± 4 mg/dL), and 30 min (105 ± 6 mg/dL). Injections of denatured insulin (*n* = 4), as vehicle control, into the LV caused no significant changes in HVGC at 5: (121 ± 3 mg/dL), 10: (118 ± 3 mg/dL), 20: (125 ± 3 mg/dL), and 30 min (119 ± 7 mg/dL). Collectively, these findings indicate that ICV insulin injection elicits a significant fall in HVGC in Wistar rats but not in SHR, as can be observed when the area under the curve of all groups were compared (Fig.1C). Furthermore, we did not observe significant changes in mean arterial pressure or heart rate after ICV administration of insulin or denatured insulin in Wistar and SHR (data not shown).

### Decreases in hepatic venous glucose concentration elicited by centrally administered insulin in conscious Wistar rats are dependent on parasympathetic outflow

**Figure 2:**
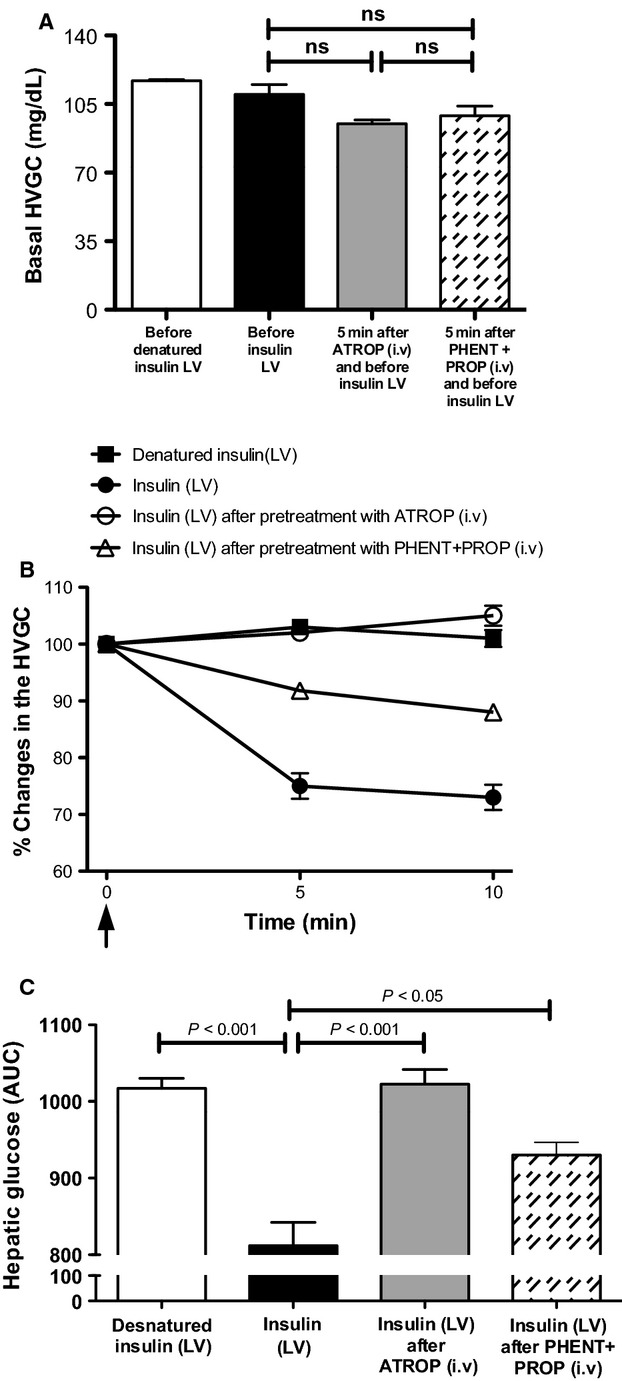
Effect of peripheral blockade of cholinergic muscarinic and *α*-, *β*-adrenergic receptors on HVGC elicited by centrally administered insulin in conscious Wistar rats. (A) Basal levels of hepatic glucose (mg/dL) prior to pretreatment with adrenergic (phentolamine; PHENT + propranolol; PROP) and cholinergic (atropine methyl-bromide; ATROP) antagonists. (B) Percentage (%) and area under the curve (AUC, panel (C) HVGC after administration of insulin (*n* = 5) and/or denatured insulin (vehicle control, *n* = 5) into the lateral ventricle of Wistar pretreated with ATROP (*n* = 5) or PHENT+PROP (*n* = 5) intravenously. One-Way ANOVA;* P* < 0.05 & *P* < 0.001. *NS* = not significant.

In order to investigate the functional relevance of the autonomic nervous system in mediating the rapid fall in HVGC, evoked by ICV injection of insulin in Wistar rats, we performed a combination of pharmacological maneuvers. To examine the role of the parasympathetic nervous system we used the muscarinic receptor antagonist, atropine methyl-bromide (ATROP) and a combination of the *α*- and *β*-adrenergic receptors antagonists, phentolamine, and propranolol (PHENT + PROP, respectively) to evaluate the role of the sympathetic nervous system. Five and 10 min before ICV injection of insulin or denatured insulin, we performed a time control experiments to measure changes in the basal HVGC in the distinct groups of animals that received an intravenous injection of ATROP or PHENT + PROP, in order to confirm that blockade of autonomic transmission *per se* would not change the baseline of HVGC (Table1). To this end, we checked and started the experiments when all groups of animals showed the same basal hepatic glucose levels (Fig.2A).

**Table 1:** Effects of autonomic neurotransmitters inhibitor agents on the baseline of hepatic venous glucose concentration

Substance Injected i.v	HVGC (mg/dL) Preinjection baseline	HVGC (mg/dL) changes from baseline	
		5 min	10 min
ATROP (6)	102 ± 3	100 ± 3	101 ± 3
PROP + PHENT (6)	106 ± 2	108 ± 6	104 ± 4

Values are means ± SE in mg/dL. (*n*) is the number of rats of each group.

The muscarinic and *α*- and *β*-adrenergic receptors antagonists were administered into the hepatic vein 10 min before the injection of insulin, or denatured insulin into the LV, an adequate time to ensure the efficacy of all autonomic antagonists (Yang et al. 1981) as previously determined by changes in the cardiovascular parameters. Blood samples were taken from hepatic vein at 5 and 10 min, times where we observed the maximum fall in HVGC after ICV injection of insulin (Fig.1B). As shown in the Figure2B, the reduction in HVGC elicited by LV insulin microinjection at 5 (84 ± 8 mg/dL) and 10 min (81 ± 3 mg/dL) compared to the basal (99 ± 5 mg/dL; *n* = 5) was completely abolished by peripheral blockade of muscarinic receptors with atropine methyl-bromide (ATROP) over the same time course (5 min: 90 ± 2 mg/dL, *P* < 0.001; and 10 min: 92 ± 3 mg/dL, *P* < 0.001; *n* = 5). Pretreatment with *α*-(PHENT) and *β*-adrenergic receptors (PROP) only partially reduced the fall in HVGC elicited by insulin at 5 (90 ± 3.5 mg/dL; *P* < 0.05; *n* = 5) and 10 min (83 ± 2.7 mg/dL; *P* < 0.05; *n* = 5) compared to basal levels (98.6 ± 4.8 mg/dL; *n* = 5). However, the fall in the HVGC observed in the animals that received the adrenergic antagonist, was also significantly different from the insulin group. These data reveal that the parasympathetic nerves exert a greater modulatory effect than the sympathetic outflow in the control of the HVGC following central insulin administration as can be observed when the area under the curve of all groups were compared (Fig.2C).

### Intracarotid injection of insulin increase SVN activity in Wistar but not in SHR

**Figure 3:**
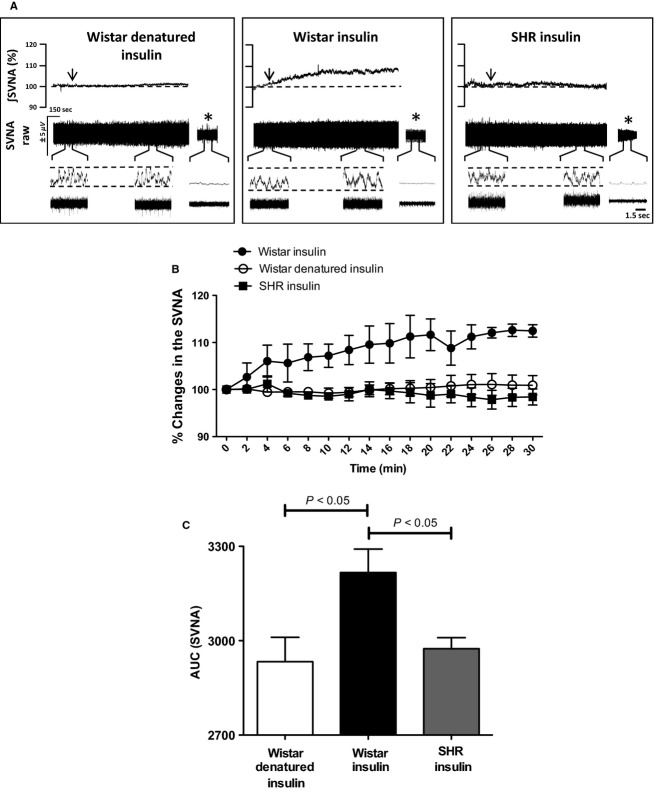
Subdiaphragmatic vagus nerve activity (SVNA) is differently modulated by intracarotid injection of insulin in Wistar and SHR. (A) Typical traces of changes in the raw (*μ*V) and integrated (∫) SVNA of one representative animal from each group after intracarotid injection of insulin (arrows). *Background noise after pump turned off. (B) Percentage (%) change and (C) area under the curve (AUC) of SVNA after intracarotid injection of insulin or denatured insulin (arrows) in Wistar (*n* = 4) and SHR (*n* = 4). One-Way ANOVA;* P* < 0.05.

To ensure that centrally acting insulin modulates HVGC by changing autonomic nerve activity, we next performed direct recordings of the vagus and sympathetic efferent nerves supplying the liver in the in situ DAPR model without the influence of anesthesia. Figure3A shows representative recording of integrated SVN activity in two different groups of animals (Wistar and SHR) that received intracarotid injection (ICA) of insulin and/or denatured insulin (control). ICA infusion of insulin in Wistar rats produced a gradual and long last increase in the SVA activity, which was significantly increased when compared to the basal values (basal: 100% vs. insulin: 112 ± 2%; *n* = 4; Fig.3B). However, ICA infusion of denatured insulin elicited no significant changes in SVN activity when compared to the baseline (basal: 100% *vs* denatured insulin: 98 ± 2%; *n* = 4; Fig.3B). Moreover, we observed that ICA infusion of insulin did not induce any significant alterations in SVN activity of SHR (basal: 100% vs. insulin: 101 ± 2%; *n* = 4, Fig.3B). These findings indicate that intracarotid infusion of insulin activates parasympathetic efferent nerves only in Wistar rats, but not in SHR (Fig.3B and C). These results are similar to those observed in the fall of hepatic venous glucose concentration evoked by ICV injection of insulin in conscious Wistar rats.

### LSSN activity is not affected by intracarotid injection of insulin in Wistar and SHR

**Figure 4:**
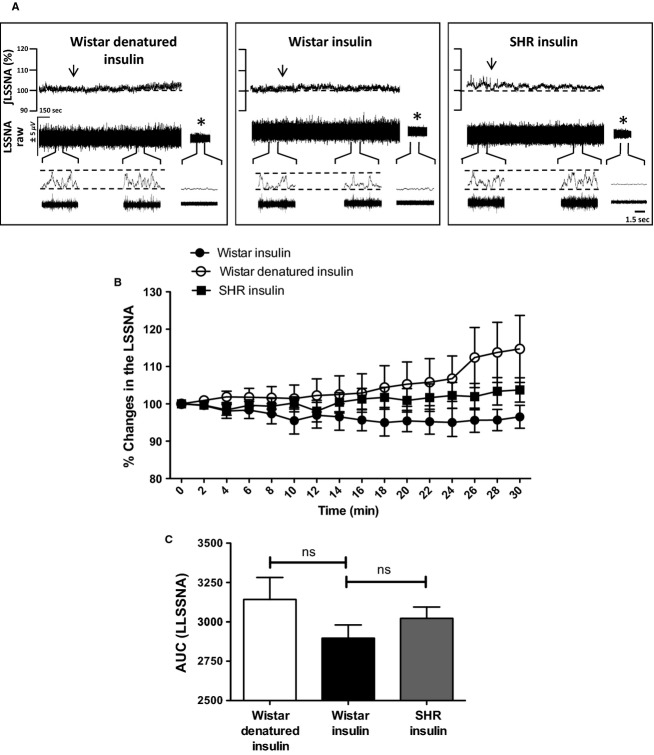
Lumbar splanchnic sympathetic nerve activity (LSSNA) is not affected by intracarotid injection of insulin in Wistar and SHR. (A) Typical traces of changes in the raw (*μ*V) and integrated (∫) LSSNA from one representative animal from each group after intracarotid injection of insulin (arrows). *Background noise after hexamethonium. (B) Percentage (%) change and (C) area under the curve (AUC) of LSSNA after intracarotid injection of insulin or denatured insulin (arrows) in Wistar (*n* = 4) and SHR (*n* = 4). One-Way ANOVA;* NS* = not significant.

Finally, we evaluated whether ICA administration of insulin might also affect LSSN activity in Wistar and SHR. Figure4A shows representative traces of LSSN activity before and after ICA administration of insulin or denatured insulin in Wistar and SHR. As can be clearly seen in Figure4B and C, there were no significant changes in LSSN activity elicited by ICA infusion of insulin in either Wistar (basal: 100% vs. insulin: 96 ± 3%, *n* = 4) or SHR (basal: 100% vs. insulin: 103 ± 3%, *n* = 4). As expected denatured insulin also failed to affect the LSSN activity when compared to baseline activity in the same group and between groups (basal: 100% vs. denatured insulin: 102 ± 8%, *n* = 4).

## Discussion

It has been well documented that changes in HGP elicited by the central actions of insulin involves the parasympathetic nervous system (Szabo et al. 1983). However, how the autonomic nervous system controls these processes in nonanesthetized rats with different sympatho-vagal balance, such as in Wistar and SHR, have not, to the best of our knowledge, been previously investigated. We have thus used two different experimental models: an in vivo conscious rat model which allowed us to determine the central actions of insulin on HVGC in an intact freely moving conscious animal, and an in situ decorticate arterially perfused rat preparation (DARP), which allowed us examine the actions of insulin on the activity of directly recorded efferent sympathetic and parasympathetic nerves.

Our main findings indicate a selective activation of hepatic vagus efferent nerve and an immediate parasympathetic-mediated decrease in HVGC elicited by central action of insulin in Wistar rats, but not SHR. It is important to emphasize that here we are measuring the hepatic venous glucose concentration, but not the hepatic glucose production (HGP) that reflects the balance across the liver over time and requires arterial and portal vein concentrations, and respective flow rates, which in fact has not been measured. In this sense, one of the goals in this study was to analyze the rapid action of insulin acting on the CNS by influencing the availability of glucose from hepatic venous circulation, but not to understand the intracellular mechanisms responsible for the provision of glucose into the circulation by the liver. For this reason we are assuming that the terminology HVGC, as a predictor index of hepatic glucose production, would be more appropriate to be used in this case.

The role of insulin, acting centrally, controlling the level of circulating glucose has received considerable attention in last decades (Szabo and Szabo 1972, 1975; Baskin et al. 1983; Szabo et al. 1983; Obici et al. 2002). Insulin is a hormone produced peripherally by the pancreas that reaches the CNS by crossing the blood–brain barrier through a saturable transport (Baura et al. 1993; Banks et al. 2000), and acts on receptors located at different brain areas, such as cortex, olfactory bulb, hypothalamus, cerebellum, and hippocampus (Havrankova et al. 1981; Plum et al. 2005). A previous study from the 1970s provided one of the first pieces of evidences that insulin can act on the CNS to regulate glucose metabolism (Szabo and Szabo 1975). At the time, the authors observed a significant decrease in systemic blood glucose after injection of insulin into the carotid artery of anesthetized normotensive rats. Later, the same authors demonstrated that a similar decrease in hepatic blood glucose could be observed after direct injection of insulin into the ventromedial hypothalamus (VMH) of anesthetized rats (Szabo et al. 1983). Taking these and other studies together (Baloyannis et al. 1987; Sakaguchi and Bray 1990) lead us to conclude that ICA administration of insulin is a viable alternative to ICV administration to test the central actions of insulin. Hence, we used this route of administration to test the central actions of insulin in the decorticated and artificially perfused rat preparation (DARP) model, which also allowed us to directly record both vagal and sympathetic nerve activities without the confounding influence of anesthetics.

Intracerebroventricular administration of insulin produces the same pattern of reduction in plasma glucose in normotensive anesthetized animals, while administration of denatured insulin or saline (as control) did not have any effects (Obici et al. 2002). Using the same paradigm, our present findings show an important role of centrally acting insulin in eliciting a significant decrease in HVGC in freely moving Wistar rats with a rapid onset (i.e., as early as 5 min after the insulin microinjection). However, it is noteworthy that here we used two experimental approaches that differ from previous studies. First, the current study was conducted in conscious intact free moving animals without the influence of anesthesia. This is an important point since studies performed in anesthetized animals typically used supraphysiological concentrations of hormones (and other drugs), moreover, anesthetics have a significant influence on cellular metabolism and sensitivity to the action of hormones (Wasserman 2009). Additionally, many anesthetics produce significant autonomic depression, which could compromise blood sugar levels as this is controlled by the autonomic nervous system (Göther 1982; Pearse 2002; Holscher et al. 2008). Another point that deserves particular attention is the concentration of insulin we used for the ICV studies, which was approximately 1000-fold lower than that used in previous studies and is therefore much closer to the physiological concentrations (Mckernan and Calaresu 1996; Obici et al. 2002; Pricher et al. 2008; Mayer et al. 2010). Thus, we believe that the combination of using unanesthetized intact animals and low, physiological doses of insulin are the best approach to evaluate the central actions of insulin and the autonomic processes that modulate HVGC.

We also demonstrated that pretreatment with the muscarinic receptors antagonist was effective at blocking the falling in the HVGC caused by ICV injection of insulin in Wistar rats. In further support of our findings, others (Szabo et al. 1983) have shown that vagotomy can blunt the decrease in HVGC induced by injection of insulin into the VMH in anesthetized rats. In addition, in the DAPR, an anesthetic-free animal model, we observed that ICA injection of insulin evoked a significant increase in the subdiaphragmatic hepatic vagus efferent nerve activity in Wistar rats but not in SHR. This increase in the efferent vagal activity could be directly related to fall in the HVGC by central action of insulin. This assertion is supported by the fact that we were able to visualize fibers from the distal common hepatic branch (where we were recording from) projecting toward the liver.

A recent study demonstrated that insulin when directly injected into the dorsal vagal complex inhibit glucose production in rats (Filippi et al. 2012). In agreement with these previous studies, the results of our recordings of SVN activity in the DAPR demonstrated that insulin activates the parasympathetic pathway and promotes a decrease in HVGC. Taken together, these data allow us to speculate that insulin receptors located in the CNS (hypothalamus and/or brainstem) evokes an increase in parasympathetic outflow to the liver resulting in a reduction in HVGC.

We were also able to show that pretreating Wistar rats with *α*- and *β*-adrenergic receptors antagonists attenuated, but did not completely blunt, the fall in HVGC after ICV injection of insulin. These data suggest that the sympathetic output might also play an important role in the control of the hepatic glucose levels. However, others (Szabo et al. 1983) have shown that adrenergic receptors antagonism does not modify the decrease in hepatic glucose following administration of insulin in the VMH. Nevertheless, we cannot neglect the involvement of sympathetic output in controlling of the HVGC via the central action of insulin, and in fact it has been showing elsewhere that ICV injection of neuropeptide Y-induced insulin resistance and changes in endogenous glucose production via activation of sympathetic output to the liver (Van den Hoek et al. 2008). However, these experiments were performed under a hyperinsulinemic-euglycemic clamp condition, which is different to our approach where we have shown a rapid effect (within 5 min) of central action of insulin by a single bolus injection into the brain and its capability to elicit an acute effect on HVGC via autonomic outflow, manly via modulation of parasympathetic output. As such, the exact sites and mechanisms of action of insulin within specific autonomic brain nuclei that control the sympatho-vagal balance, which ultimately modulates hepatic venous glucose levels, need to be addressed.

As for the experiments performed in the DARP model, we have not observed any change in LSSNA after insulin injection ICA, despite have shown an attenuation of the fall in HVGC after insulin injection ICV. An possible explanation for this discrepancy, may be in part reconciled, based on the fact that we are looking at the central action of insulin upon the sympathetic outflow and hepatic venous glucose concentration from two different perspectives: (1) in the DARP by direct monitoring of sympathetic nerve activity to a specific region; (2) and in conscious rats by indirect inference of the sympathetic modulation on HVGC via pharmacological manipulation, that is, by using adrenoceptor's antagonists. In relation to the pharmacological approach we are using nonspecific and broad-spectrum adrenergic antagonists to block the adrenoceptors located at all organs. In this case, the adrenergic antagonists are not selective for one specific territory of interest, such as liver, and they can also affect other organs, such as pancreas that is involved in glucoregulatory hormone release, which in turn control the glucose metabolism. On the other hand, as for the nerve recordings of the sympathetic branch (in the DARP) we are looking at a more specific territory, although for the splanchnic sympathetic activity, which is a multiunit recording of postganglionic inputs not only exclusive to the liver, but also to GI tract, pancreas and other organs. In this sense, we could argue that from the splanchnic sympathetic branch only few fibers that exclusively innervate the liver could be firing, but we are not observing any effect on the total LSSNA because we are performing multifibers recordings, and the sympathetic tone of the whole nerve could swamp the low activity of those fibers going directly to the liver. Perhaps, if would be possible to record the sympathetic fibers that exclusively innervate the liver we might be able to observe changes in its activity due to central action of insulin. However, due to the complexity and specific anatomical organization of hepatic nerves, selective efferent nerve recordings for a single visceral organ, borders on the impossible (Berthoud 2004).

As mentioned earlier, the same dose of insulin used in Wistar rats affected neither hepatic venous glucose levels nor the SVN activity in SHR group. At this stage we are not able to explain the discrepancy found in these two strains. However, we cannot rule out the possibility that SHR exhibits central insulin resistance, which is a well-known feature of this animal (Hulman et al. 1993). Interestingly, a lower abundance of central insulin receptors have been reported in the brain nuclei involved in modulating autonomic control (Baskin et al. 1987; Banks et al. 2000). Several studies have shown that the SHR is insulin resistant, hyperinsulinemic and has sympathetic hyperactivity (Trippodo and Frohlich 1981; Brands and Hall 1992; Secchi et al. 1996). Some authors have reported that expression of insulin receptors was decreased in both the liver and kidneys of SHR, providing a possible explanation for the insulin resistance (Secchi et al. 1996). Moreover, SHRs have been shown to be insulin resistant when compared to Wistar-Kyoto using a hyperinsulinemic/euglycemic clamp approach (Hulman et al. 1993).

We chose to use SHR aged 8–9 weeks that do not yet have elevated blood pressures, but present with sympathetic hyperactivity (Cabassi et al. 1998) to avoid a possible influence of high blood pressure effects on the activation of cardiovascular reflex feedback mechanisms (such as baroreflex buffering the autonomic outflow). However, what remains unclear is whether the sympathetic hyperactivity observed in this strain at the prehypertensive stage is more prominent for certain target organs, such as the liver.

Taken together, our findings show that the central actions of insulin plays a potentially very important role in controlling hepatic venous glucose concentration mainly by activation of parasympathetic nerves to the liver of Wistar but not in SHR. Moreover, we can postulate that autonomic imbalance or insulin resistance in SHR (or a combination of these) might contribute to the defective central action of insulin in control of HVGC. Thus, a better understanding of the role of the autonomic nervous system in regulating hepatic glucose levels by the central actions of insulin might help to uncover new pharmacological and potentially nonpharmacological therapies to treat diseases such as hypertension and diabetes.

## References

[b1] Antunes VR, Yao ST, Pickering AE, Murphy D, Paton JF (2006). A spinal vasopressinergic mechanism mediates hyperosmolality-induced sympathoexcitation. J. Physiol.

[b2] Aronoff SL, Berkowitz K, Shreiner RN, Want L (2004). Glucose metabolism and regulation: beyond insulin and glucagon. Diabetes Spectrum.

[b3] Baloyannis SJ, Theoharides TC, Manolides LS (1987). Synaptic alterations in the acoustic córtex of the rat following insulin-induced hypoglycemia. Arch Otorhinolaryngol.

[b4] Banks WA, Faar SA, Morley JE (2000). Permeability of the blood-brain barrier to albumin and insulin in the young and aged SAMP8 Mouse. J. Gerontol. A Biol. Sci. Med. Sci.

[b5] Baskin DG, Guest D, Porte K, Dorsa DM (1983). Insulin in the brain. Ann. Rev. Physiol.

[b6] Baskin DG, Fliglewicz DP, Woods SC, Dorsa D, Porte DM (1987). Insulin in the brain. Ann. Rev. Physiol.

[b7] Baura GD, Foster DM, Kahn D, Porte SE, Bergman RN, Cobelli C (1993). Saturable transport of insulin from plasma into the central nervous system of dogs in vivo. J. Clin. Investig.

[b8] Berthoud HR (2004). Anatomy and function of sensory hepatic nerves. Anat. Rec.

[b9] Berthoud HR, Kressel M, Neuhuber WL (1992). An anterograde tracing study of the vagal innervation of rat liver, portal vein and biliary system. Anat. Embryol. (Berl).

[b10] Brands MW, Hall JE (1992). Insulin resistance, hyperinsulinemia, and obesity-associated hypertension. J. Am. Soc. Nephrol.

[b11] Cabassi A, Calzolari M, Bruschi G, Borghetti A (1998). Regional sympathetic activity in pre-hypertensive phase of spontaneously hypertensive phase of spontaneously hypertensive rats. Life Sci.

[b12] Filippi BM, Yang CS, Tang C, Lam KJ (2012). Insulin activates Erk1/2 signaling in the dorsal vagal complex to inhibit glucose production. Cell Metab.

[b13] Gerozissis K (2003). Brain insulin: regulation, mechanisms of action and functions. Cell. Mol. Neurobiol.

[b14] Göther M (1982).

[b15] Havrankova J, Browntein M, Roth J (1981). Insulin and insulin receptors in rodent brain. Diabetologia.

[b16] Holscher C, Aalten LV, Sutherland C (2008). Anaesthesia generates neuronal insulin resistance by inducing hypothermia. BMC Neurosci.

[b17] Hulman S, Falkner B, Freyvogel N (1993). Insulin resistance in the conscious spontaneously hypertensive rat: euglycemic hyperinsulinemic clamp study. Metabolism.

[b18] Mayer MA, Giani JF, Hocht C, Silberman EA, Muños MC, Taira CA (2010). Centrally administered insulin potentiates the pressor response to angiotensin II. Regul. Pept.

[b19] Mckernan AM, Calaresu FR (1996). Insulin microinjection into the nucleus tractus solitarii of the rat attenuates the baroreceptor reflex. J. Auton. Nerv. Syst.

[b20] Morgan DA, Balon TW, Ginsberg BH, Mark AL (1993). Nonuniform regional sympathetic nerve responses to hyperinsulinemia in rats. Am. J. Physiol.

[b21] Nordlie RC, Foster JD, Nordlie RC, Foster JD, Lange AJ (1999). Regulation of glucose production by the liver. Annu. Rev. Nutr.

[b22] Obici S, Zhang BB, Karkanias G, Rosseti L (2002). Hypothalamic insulin signaling is required for inhibition of glucose production. Nat. Med.

[b23] Paxinos G, Watson C (2007). The rat brain in stereotaxic coordinates.

[b24] Pearse W (2002). The cardiovascular autonomic nervous system and anaesthesia. S Afr. J. Anaesth. Anal.

[b25] Perseghin G, Regalia E, Battezzati A, Vergani S, Pulvirenti A, Terruzzi I (1997). Regulation of glucose homeostasis in humans with denervated livers. J. Clin. Invest.

[b26] Phillips RJ, Baronowsky EA, Powley TL (1997). Afferent innervation of gastrointestinal tract smooth muscle by the hepatic branch of the vagus. J. Comp. Neurol.

[b27] Pilkis SJ, Granner DK (1992). Molecular physiology of the regulation of the hepatic gluconeogenesis and glycolysis. Annu. Rev. Physiol.

[b28] Plum L, Schubert M, Bruning J (2005). The role of insulin receptor signaling in the brain. Trends Endocrinol. Metabol.

[b29] Pocai A, Lam TK, Gutierrez-Juarez R, Obici S, Schwartz GJ, Bryan J (2005). Hypothalamic K(ATP) channels control hepatic glucose production. Nature.

[b30] Potenza MA, Marasciulo FL, Chieppa DM, Brigiani GS, Formoso G, Quon MJ (2005). Insulin resistance in spontaneously hypertensive rats is associated with endothelial dysfunction characterized by imbalance between NO and ET-1 production. Am. J. Physiol. Heart Circ. Physiol.

[b31] Pricher MP, Freeman KL, Brooks VL (2008). Insulin in the brain increases gain of baroreflex control of heart rate and lumbar sympathetic nerve activity. Hypertension.

[b32] Sakaguchi T, Bray GA (1990). Ventromedial hypothalamic lesions attenuate responses of sympathetic nerves to carotid arterial infusion of glucose and insulin. Int. J. Obes.

[b33] Secchi LA, Griffin CA, Giacchetti G, Zingaro L, Catena C, Bartoli E (1996). Abnormalities of insulin receptors in spontaneously hypertensive rats. Hypertension.

[b34] Szabo O, Szabo AJ (1972). Evidence for an insulin-sensitive receptor in the central nervous system. Am. J. Physiol.

[b35] Szabo A, Szabo AJ (1975). Studies on the nature and mode of action of the insulin- sensitive glucoregulator receptor in the central nervous system. Diabetes.

[b36] Szabo AJ, Iguchi A, Burlesson PD, Szabo O (1983). Vagotomy or atropine blocks hypoglycemic effect of insulin injected into ventromedial hypothalamic nucleus. Am. J. Physiol. Endocrinol. Metabol.

[b37] Trippodo NC, Frohlich ED (1981). Similarities of genetic (spontaneous) hypertension, men and rat. Circ. Res.

[b38] Van den Hoek AM, Heijningen C, Schröder-van der Elst JP, Ouwens DM, Havekes LM, Romijn JA (2008). Intracerebroventricular administration of neuropeptide Y induces hepatic insulin resistance via sympathetic innervation. Diabetes.

[b40] Wasserman DH (2009). Four grams of glucose. Am. J. Physiol. Endocrinol. Metab.

[b41] Yang JW, Raizada MK, Fellows RE (1981). Effects of insulin on cultured rat brain cells: stimulation of ornithine decarboxylase activity. J. Neurochem.

